# Surfactant-Triggered Molecular Gate Tested on Different Mesoporous Silica Supports for Gastrointestinal Controlled Delivery

**DOI:** 10.3390/nano10071290

**Published:** 2020-06-30

**Authors:** Elisa Poyatos-Racionero, Isabel González-Álvarez, Marta González-Álvarez, Ramón Martínez-Máñez, M. Dolores Marcos, Andrea Bernardos, Elena Aznar

**Affiliations:** 1 CIBER de Bioingeniería, Biomateriales y Nanomedicina (CIBER-BBN), 46022 Valencia, Spain; elpora@upvnet.upv.es (E.P.-R.); rmaez@qim.upv.es (R.M.-M.); elazgi@upvnet.upv.es (E.A.); 2 Instituto Interuniversitario de Investigación de Reconocimiento Molecular y Desarrollo Tecnológico (IDM), Universitat Politècnica de València, Universitat de València. Camino de Vera s/n, 46022 Valencia, Spain; 3 Departamento de Ingeniería, Sección de Farmacia y Tecnología Farmacéutica, Universidad Miguel Hernández., 03550 Alicante, Spain; isabel.gonzalez@umh.es (I.G.-Á.); marta.gonzalez@umh.es (M.G.-Á.); 4 Unidad Mixta de Investigación en Nanomedicina y Sensores, Universitat Politècnica de València, Instituto de Investigación Sanitaria La Fe, Av Fernando Abril Martorell 106, 46026 Valencia, Spain; 5 Unidad Mixta UPV-CIPF de Investigación en Mecanismos de Enfermedades y Nanomedicina, Universitat Politècnica de València, Centro de Investigación Príncipe Felipe, C/ Eduardo Primo Yúfera 3, 46100 Valencia, Spain; 6 Departamento de Química, Universitat Politècnica de València, Camino de Vera s/n, 46022 Valencia, Spain

**Keywords:** mesoporous silica, oleic acid, molecular gate, MCM-41, MCM-48, SBA-15, UVM-7, controlled release, kinetic modelling, gastrointestinal delivery

## Abstract

In recent decades, the versatility of mesoporous silica particles and their relevance to develop controlled release systems have been demonstrated. Within them, gated materials able to modulate payload delivery represent great advantages. However, the role played by the porous matrix in this kind of systems is scarce. In this work, different mesoporous silica materials (MCM-41, MCM-48, SBA-15 and UVM-7) are functionalized with oleic acid as a molecular gate. All systems are fully characterized and their ability to confine the entrapped cargo and release it in the presence of bile salts is validated with release assays and in vitro digestion experiments. The cargo release profile of each synthesized support is studied, paying attention to the inorganic scaffold. Obtained release profiles fit to Korsmeyer–Peppas model, which explains the differences among the studied supports. Based on the results, UVM-7 material was the most appropriate system for duodenal delivery and was tested in an in vivo model of the Wistar rat. Payload confinement and its complete release after gastric emptying is achieved, establishing the possible use of mesoporous silica particles as protection and direct release agents into the duodenum and, hence, demonstrating that these systems could serve as an alternative to the administration methods employed until now.

## 1. Introduction

The use of mesoporous silica particles (MSPs) as delivery systems of bioactive molecules has been reinforced during recent years. The great loading capacity in their ordered pore matrix, low toxicity levels and high stability in biological conditions, especially when organic moieties are attached to the external surface [1,2,3], make them ideal supports for passive release supports. In these systems, cargo molecules are released from the pores of the particles by diffusion mechanisms [4]. Moreover, MSPs also become ideal candidates for active (or controlled) release, where a stimulus-triggered mechanism regulates cargo delivery [4,5]. The combination of a suitable pore size, together with an appropriate external functionalization to control payload delivery [6], gives them versatile uses in different fields, such as catalysis [7,8], controlled drug release [9,10,11,12,13], sensing [14,15,16,17], communication [18,19] and protection and controlled administration of molecules with nutraceutical interest [20,21,22,23].

Within the wide field of MSPs, microparticles present a special interest for the controlled release in the lumen of the gastrointestinal tract (GIT), since their large size makes their internalization in healthy epithelial tissues difficult [24,25,26]. In the field of MSPs, it is possible to find a wide diversity of structures with different morphology, composition or pore pattern [27,28]. Despite this wide range and versatility, the influence of inorganic support on the cargo release has been only thoroughly examined in passive release systems [4,29,30,31]. Generally, on active release works, the triggered opening of the molecular gate is the main pursued objective with the aim of minimizing the non-specific payload release. Studies of active release in which the importance of inorganic support is evaluated are rarely found [32,33,34]; however, the release process can strongly depend on the utilized support. 

Another aspect to be considered in delivery systems based on MSPs is that sustained release over time is usually the most common objective in drug administration into the organism, in order to promote the progressive incorporation of the components into the blood stream [35]. However, this statement, although true for many substances, is not applicable to the entire spectrum of drugs currently administered. There are drugs that require different action times, from an overtime sustained activity to an immediate one, or even the ability to combine both sustained and immediate action depending on the organism requirements, as in the case of insulin [36]. There are also processes in which a massive release is needed after a protection stage, such as intestinal absorption after protection from the stomach environment. This is the case of patients with gastric problems, or drugs that suffer from gastric malabsorption because of pH or the enzymes present in this organ. 

Slightly detailing these last two examples, several drawbacks are found. On the one hand, patients with severe gastric diseases such as ulcers, bariatric surgery or gastrectomy undergo significant modifications of the stomach pH or the reconstructed area [37], as well as a variation of the intestinal microbiota [38]. The aforementioned aspects highly influence the patient administration of medications that require strict pH conditions for their correct absorption [39,40]. On the other hand, there are drugs that suffer from degradation by the pH of the gastric juice and the exposition time to the enzymes secreted in the gastric and intestinal mucosa [41]. Due to these inconveniences, the direct intraduodenal perfusion is selected in some cases as an alternative administration route. With this system, the immediate absorption of the drug in blood is achieved and concentration fluctuations are decreased [42,43]. However, this kind of administration is dependent on surgery and on an intraduodenal pump that injects the selected drug continuously [44,45]. 

In cases like these, an immediate release system in the duodenum would be the recommended solution to protect the drug and the stomach in equal parts. Additionally, with this solution, the dose of bioactive molecule that will reach the duodenum will not be reduced. These facts show that different modes of release could be necessary, and the possibility of having different systems that deliver their cargo in a sustained, immediate or combined way is an important aspect to be considered.

In this context, the objective of this research focuses on the controlled release performance of a chosen molecular gate, equally effective when it is functionalized on different MSPs, and on the role of the several supports in managing payload delivery. Thus, the release process in different inorganic structures perfectly protected by the same capping organic moiety would be determined by the properties of the loaded molecule and the material’s pore structure. This fact allows one to have a set of microdevices with perfectly defined release profiles which enable the selection of the ideal one for a desired application.

Oleic acid (OA) has been demonstrated to be a good gating entity when functionalized onto MSPs for cargo delivery in the small intestine, avoiding cargo release when no bile salts (or surfactants) are present [46]. Its good behavior in both open and closed state was the reason why it was chosen as model to act as the molecular gate for the different mesoporous systems under this study. The main objective of this work is to survey the release profiles of different MSPs capped with OA in the presence of bile salts with the aim of finding the best one for cargo protection from the adverse conditions of the stomach and maximum release into the duodenum.

## 2. Materials and Methods 

### 2.1. Chemicals and Cell Culture Media

Tetraethylortosilicate (TEOS), triethanolamine (TEAH_3_), sodium hydroxide (NaOH), N-cetyltrimethylammonium bromide (CTAB), Pluronic 123 (P123), hydrochloric acid 37% (HCl), aqueous ammonia 28% (NH_4_OH), trifluoroacetic acid (TFA), (3-aminopropyl)triethoxysilane (APTES), *N*-(3-dimethylaminopropyl)-*N*′-ethylcarbodiimide (EDC), rhodamine B (RhB), oleic acid (OA) and all the chemicals for the digestive fluids (bile salts included) were provided by Sigma (Sigma-Aldrich Química S.L., Madrid, Spain). Ethanol (extra pure), dimethyl sulfoxide (DMSO), acetonitrile (ACN) and acetone were purchased from Scharlab (Scharlab S.L., Barcelona, Spain). 3-(4,5-dimethylthiazol-2-yl)-2,5-diphenyltetrazolium bromide (MTT) was provided by ThermoFisher (Thermo Fisher Scientific Inc., Madrid, Spain). Phosphate Buffer Solution (PBS), Dulbecco’s Modified Eagle’s Medium (DMEM), Fetal Bovine Serum (FBS), penicillin/streptomycin antibiotic (P/S), non-essential amino acids and all the needed mediums for cell culture were provided by Labclinics (Labclinics S.A., Barcelona, Spain).

### 2.2. Mesoporous Silica Particles Synthesis

MCM-41 microparticles (M41) were synthesized following the “atrane route” according to the method described by Cabrera et al. [47]. CTAB was used as the structure-directing agent, and the molar ratio of the reagents was fixed to 7 TEAH_3_:2 TEOS:0.52 CTAB:0.5 NaOH:180 H_2_O. CTAB was added to a solution of TEAH_3_ containing NaOH and TEOS at 118 °C. After dissolving CTAB in the solution, water was slowly added with vigorous stirring at 70 °C. The resultant white suspension formed after a few minutes was stirred for 1 h at room temperature, and then aged in an autoclave at 100 °C for 24 h.

MCM-48 microparticles (M48) were made applying the method described by Meléndez-Ortiz [48]. The molar ratio of the reagents was 0.11 CTAB:100 H_2_O:13.1 EtOH:1.3 NH_4_OH:0.2 TEOS. CTAB was dissolved in a mixture of H_2_O:EtOH, and then NH_4_OH was poured. Then, TEOS was immediately added into the solution under vigorous stirring for 16 h at room temperature.

SBA-15 (S15) microparticles were synthesized following the method described by Zhao et al. [49]. P123 was used as a template of the mesoporous structure. The molar ratio of the reagents was 0.017 P123:1.0 TEOS:6 HCl:196 H_2_O. For the microparticle synthesis, an aqueous solution of 4 g of P123 was mixed with HCl 37%, and this liquor was stirred for 2 h, after which the silica source (TEOS) was added dropwise. This final mixture was stirred for a further 24 h, and it was aged in an autoclave at 100 °C for another 24 h.

UVM-7 particles (U7) were synthesized following the method presented by El Haskoury et al. [50], based also on the “atrane route”. The molar ratio of the reagents was fixed at 7 TEAH_3_:2 TEOS:0.52 CTAB:180 H_2_O. The TEAH_3_/TEOS mixture was heated to 150 °C in a Dean–Stark apparatus until no condensation of ethanol was observed. The reaction was cooled to 90 °C and CTAB was gradually added in small portions, followed by water addition. The reaction was aged for 24 h at room temperature.

In all synthesis, the resulting powder was isolated from the mother liquor by filtration (M41, M48 and S15) or by centrifugation (U7), washed with deionized water until neutral pH was achieved and dried at 70 °C overnight. The final mesoporous materials were obtained by calcination of the as-synthesized solids at 550 °C for 5 h in an oxidant atmosphere with the aim of removing the surfactant template.

### 2.3. Synthesis of Solids Loaded with RhB and Capped with OA

Solids were synthesized following the procedure described in previous works of the group with slight modifications [46]. In a typical synthesis, 500 mg of the initial materials (M41, M48, S15 or U7) were suspended in 15 mL of an aqueous solution of RhB (192 mg, 0.4 mol) and stirred during 24 h to obtain the loaded solids (M41-RhB, M48-RhB, S15-RhB and U7-RhB, respectively), which were centrifuged and dried under vacuum.

In the next step, 500 mg of the loaded solids were suspended in 20 mL of ethanol, and an excess of APTES (2.34 mL, 10 mmol) was added to the previous mixture. The reaction was stirred for 5.5 h to obtain the APTES-functionalized solids (M41-APTES, M48-APTES, S15-APTES and U7-APTES), which were isolated by centrifugation and dried under vacuum.

To obtain the final solids, 500 mg of the APTES-functionalized solids were added to a solution of EDC (50 mg) and OA (5 mL) previously dissolved in 30 mL of ethanol. The reaction was stirred at room temperature during 24 h. The final solids, functionalized with OA (M41-OA, M48-OA, S15-OA and U7-OA, respectively, or MSP-OA in general), were isolated by centrifugation, washed with a mixture of EtOH:H_2_O (1:2) and dried at 37 °C for 24 h.

### 2.4. Methods Used for Characterization

Powder X-ray diffraction (XRD), transmission electron microscopy (TEM), N_2_ adsorption–desorption isotherms, FTIR, thermogravimetric analysis (TGA) and fluorescence spectroscopy were employed to characterize the synthesized solids. XRD was performed on a Bruker D8 Advance diffractometer (Bruker, Coventry, UK) using Cu Kα radiation. TEM images were obtained with a JEOL JEM-1010 (JEOL Europe SAS, Croissysur-Seine, France). N_2_ adsorption–desorption isotherms were recorded with a Micromeritics TriStar II Plus automated analyzer (Micromeritics Instrument Corporation, Norcross, GA, USA). The samples were degassed at 120 °C in vacuum overnight. The specific surface areas were calculated from the adsorption data in the low-pressure range using the BET model. Pore size was determined by following the BJH method.

The functionalization process of all the solids was followed by infrared spectroscopy in a Bruker Tensor 27 FTIR spectrometer. The composition of loaded and functionalized particles was determined by TGA. Thermogravimetric analyses were carried out on a TGA/SDTA 851e Mettler Toledo balance (Mettler Toledo Inc., Schwarzenbach, Switzerland) using an oxidant atmosphere (air, 80 mL/min) with a heating program consisting of a heating ramp of 10 °C per minute from 393 to 1273 K and an isothermal heating step at this temperature of 30′. Fluorescence spectroscopy was carried out on a JASCO FP-8300 Spectrofluorometer (JASCO, Easton, OH, United States).

### 2.5. Cargo Delivery

In a typical experiment, 5 mg of each final solid (M41-OA, M48-OA, S15-OA and U7-OA respectively) were placed in 10 mL of PBS (pH 7.5) as general aqueous fluid, and 10 mL of bile salts suspension (100 mg of bile salts in 10 mL of PBS, pH 7.5), simulating and simplifying the conditions at the duodenum. At certain times aliquots were taken and filtered. The delivery of RhB from the pore voids to the different solutions was analyzed via the fluorescence emission band of this molecule at 572 nm (excitation at 555 nm).

### 2.6. Cargo Release Kinetics

The payload release kinetics from the pore system of all the inorganic supports were calculated using different mathematical models. The employed models are [51]:

Zero order: *Cargo release* (%) = *k*_0_ · *t* (1)

First order: *log Cargo release* (%) = *log C*_0_ + (*k*_1_ · *t*)/2.303 (2)

Higuchi: *Cargo release* (%) = *K_H_* · *t*^1/2^ (3)

Korsmeyer–Peppas: *log Cargo release* (%) = *K* · *t^n^* + *b* (4)

### 2.7. In Vitro Digestion

An in vitro digestion was performed to all the solids according to the method described by Versantvoort et al. [52]. The procedure was followed to simulate the chemical composition and pH of the different gastrointestinal (GI) steps (mouth, stomach and duodenum). The residence times of each organ were also based on this protocol. The pH of the fluids (saliva pH 7.5, gastric fluid pH 1.5–2, duodenal fluid pH 7.8–8) was checked and adjusted to the appropriate value when necessary with NaOH (1M) or HCl (37%).

### 2.8. Cell Culture Conditions

Caco-2 human colon adenocarcinoma cells were grown in DMEM medium supplemented with 10% FBS, 1% P/S and 1% non-essential amino acids. Cells were maintained at 37 °C in an atmosphere of 5% CO_2_ and 95% air and underwent passage when 80% confluence was reached.

### 2.9. MTT Cell Viability Assay

Caco-2 cells were cultured in sterile 96-well plates at density of 2 × 10^4^ cells/well and were incubated 24 h in the atmosphere conditions previously reported. Then, all the tested MSPs were suspended in final concentrations and each one was tested in 8 wells; control cells were absent of any MSP. After 24 h of incubation, cells were washed with PBS in order to remove MSPs and then 100 μL of MTT solution in non-supplemented DMEM medium (0.5 mg/mL) were added to each well, and the plates were incubated for another 2 h. After incubation, supernatant DMEM-MTT medium was removed, and 100 μL of DMSO were added to each well and the plate was softly shacked until complete solution of formazan crystals to ensure homogeneous distribution of violet color. Then, the absorbance was measured at λ_exc_ = 550 nm. The reported results are given as average of the results of two independent experiments.

### 2.10. In Vivo Pharmacokinetic Studies

To assess the in vitro results, in vivo experiments with U7-OA (which showed the best performance in the in vitro studies) in rats were carried out and RhB concentration in plasma was studied. The experimental protocol was approved by the Ethics Committee for Animal Experimentation of the University in accordance with the 2010/63/EU directive of 22 September 2010 (Spain, code A1330354541263). A cannula was implanted in the jugular vein 24 h before administration to take blood samples (0.5 mL). A dose of 2 mL of RhB solution (0.84 mg/mL) was administrated orally in group 1 and the same dose of a suspension of U7-OA (containing 150 mg of solid which releases 1.67 mg of RhB, in 2 mL) was administered in group 2. Blood samples were withdrawn at 15′, 30′, 45′, 1 h, 1.5 h, 2 h, 3 h, 4 h, 5 h, 6 h, 7 h and 8 h and plasma was immediately separated by centrifugation (10,000 rpm for 10′) and then proteins were precipitated with methanol (4 °C). Samples were analyzed by HPLC with Novapack C18 (Waters Alliance® e2695, Milford, MA, USA), mobile phase ACN:H_2_O 30:70 *v*/*v* with 1% of TFA and fluorescence detector using λ_exc_ = 555 and λ_em_ = 572 nm. 200 mL of volume injection were used and a flow of 1 mL/min was fixed. The method was previously validated with adequate linearity, precision and accuracy (R > 0.99 and coefficient of variation < 5%)

## 3. Results and Discussion

### 3.1. Design, Synthesis and Characterization of Solids

In the present project, RhB was encapsulated into different mesoporous silica materials, which were finally capped with OA. The capacity of OA to effectively cap the pores and the ability of the materials to deliver the cargo in different conditions were evaluated. Four different porous silica supports (MCM-41, MCM-48, SBA-15 and UVM-7) with similar micrometric particle size, but with different shape, pore size and pore distribution were subjected to study. In this work, the cargo release mode of the different microdevices was evaluated in the presence of bile salts, which act as emulsifiers. The action mechanism of OA as a molecular gate was described in previous works of the group [46]. The interaction forces between the hydrophobic tails of the lipid molecules anchored to the MSPs surface act as closing forces, which avoid cargo release from the system to an aqueous environment. When surfactant molecules, like bile salts, are present in the particles’ surroundings, this interaction is interrupted, and the encapsulated payload is then allowed to diffuse.

**Figure 1:**
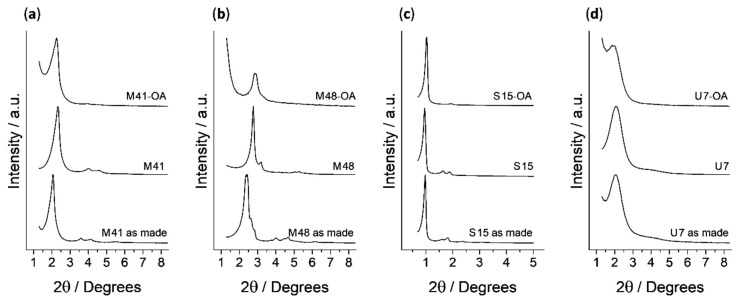
Normalized powder X-ray patterns of all the synthesized solids. From left to right, (**a**) MCM-41, (**b**) MCM-48, (**c**) SBA-15 and (**d**) UVM-7 materials. From bottom to top: as made, calcined, and the loaded and functionalized final material (MSP-OA), respectively.

The synthesized solids were characterized using usual techniques. Normalized powder X-ray patterns of all solids (as made, calcined, loaded and functionalized) are shown in Figure 1. The observed changes in the shapes of peaks and positions in the diffractograms were independently evaluated for each mesoporous material. The MCM-41 solids’ diffractograms show four low-angle peaks characteristic of a hexagonal arrangement, indexed from left to right as (1 0 0), (1 1 0), (2 0 0) and (2 1 0) Bragg reflections, respectively. It can be observed that the (1 0 0) peak is notably shifted to higher 2θ values in the calcined material curve (M41) with respect to the as made one. This shift is related to cell contraction due to the condensation of silanols during the calcination process. Finally, it can also be observed that, in the curves corresponding to the final solid (M41-OA), the reflections (1 1 0), (2 0 0) and (2 1 0) have almost disappeared because of a decrease in the contrast due to the external functionalization and pore filling. However, the clear presence of the (1 0 0) peak at all synthesis stages demonstrates that neither calcination nor pore filling followed by external functionalization modify the material’s mesoporous structure. All these arguments are also applicable to the materials based on the structures of SBA-15 and UVM-7, with slight differences. On the one hand, SBA-15 materials exhibit peaks narrower than the MCM-41 ones due to a more ordered structure and smaller 2θ values corresponding to a larger unit cell related to their larger pore size. On the other hand, the diffractograms of the UVM-7 family solids are broader than those of the aforementioned materials due to the less ordered structure of their pore matrix. The different porous structure presented by solids based on the MCM-48 material makes the peaks of their diffractograms markedly different from those previously detailed. MCM-48 peaks positions are in agreement with the values reported already in literature, and, assuming a cubic three-dimensional mesostructure, the main peaks for MCM-48 can be indexed as (2 1 1), (2 2 0), (3 2 1), (4 2 0) and (3 3 2). Moreover, displacement of peaks positions to higher values after calcination and the order loss after loading and functionalization processes are phenomena that also occur with this material.

**Figure 2:**
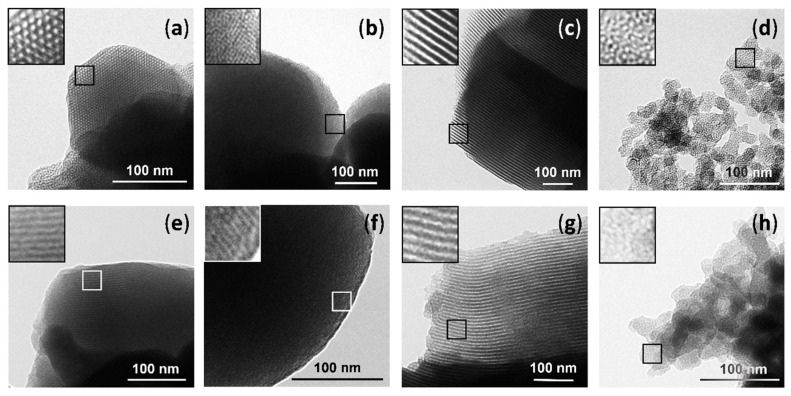
TEM images of calcined solids (top) (**a**) M41, (**b**) M48, (**c**) S15, (**d**) U7 and loaded and functionalized solids (bottom) (**e**) M41-OA, (**f**) M48-OA, (**g**) S15-OA, (**h**) U7-OA. Insets correspond to a 3× magnification of the respective selected zones.

The presence of the mesoporous structure in the final functionalized solids was also confirmed by TEM analysis. Typical pores and channels of the MSP matrix are visualized as alternate black and white stripes (see Figure 2, top) or white circles, even with the loss of contrast produced by the presence of organic matter in loaded and functionalized solids (see Figure 2, bottom). In the case of the UVM-7, the different intraparticle structure of this material that can be considered as made of fussed irregular mesoporous nanoparticles, giving rise to a textural-intraparticle secondary pore system, can also be observed in Figure 2 (d and h).

**Figure 3:**
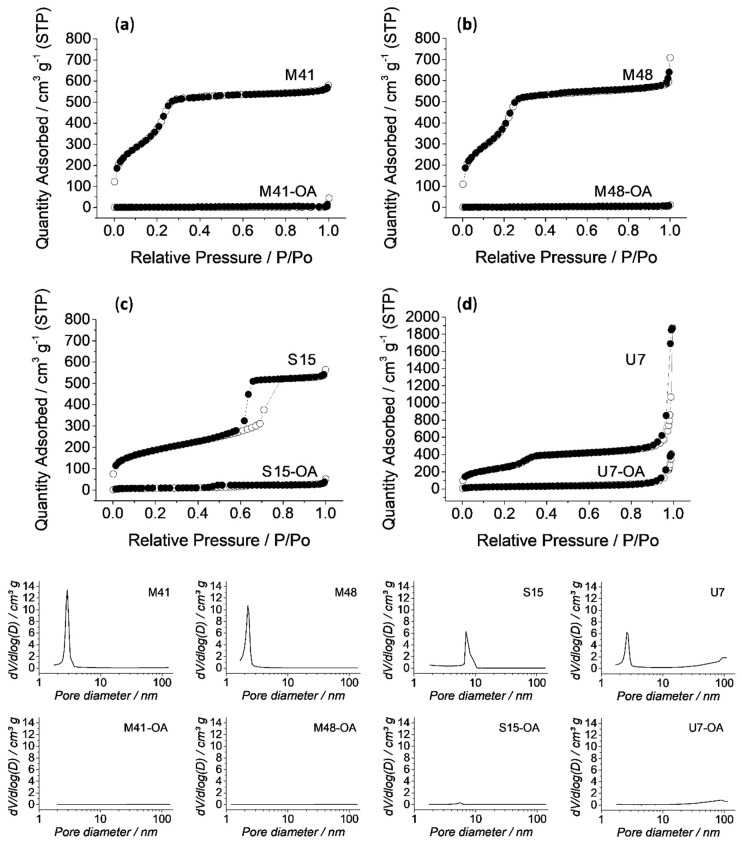
Top: Nitrogen adsorption (○)–desorption (**●**) isotherms for all the calcined and final solids (**a**) MCM-41, (**b**) MCM-48, (**c**) SBA-15, (**d**) UVM-7. Bottom: Pore distribution graphs of all the calcined and final solids (MSP-OA), indicated in its own graph.

The N_2_ adsorption–desorption isotherms of the calcined microparticles are shown in Figure 3. A typical curve for these mesoporous solids consisting of an adsorption step at intermediate P/P_0_ value (0.1–0.3) can be observed. These curves correspond to a type IV isotherm, in which the produced step deals with nitrogen condensation inside the mesopores of each structure. The absence of a hysteresis loop in this interval in MCM-41, MCM-48 and UVM-7 structures and the narrow BJH pore distribution (Figure 3, bottom), suggest the existence of uniform cylindrical mesopores. The presence of hysteresis loop in SBA-15 solids indicates adsorption and desorption processes carried out in different ways, which is related with the high pore size of this material. Furthermore, it is important to point out that the large N_2_ adsorption at high P/P_0_ values for UVM-7 material correspond to the filling of the textural intraparticle pores.

The N_2_ adsorption–desorption isotherm of MSP-OA solids is typical of mesoporous systems with practically filled mesopores (see Figure 3). For all the loaded and functionalized solids, the N_2_ adsorbed is negligible due to the inability of the gas to condense into the blocked pores.

Consequently, relatively low N_2_ adsorbed volume and surface area (see Table 1) values were calculated. In fact, these solids show flat curves when compared to those of the calcined parent material, which indicates significant pore blocking and the subsequent absence of appreciable mesoporosity. Only in the case of the U7-OA material, some textural-intraparticle porosity is maintained after loading and functionalization processes.

**Figure 4:**
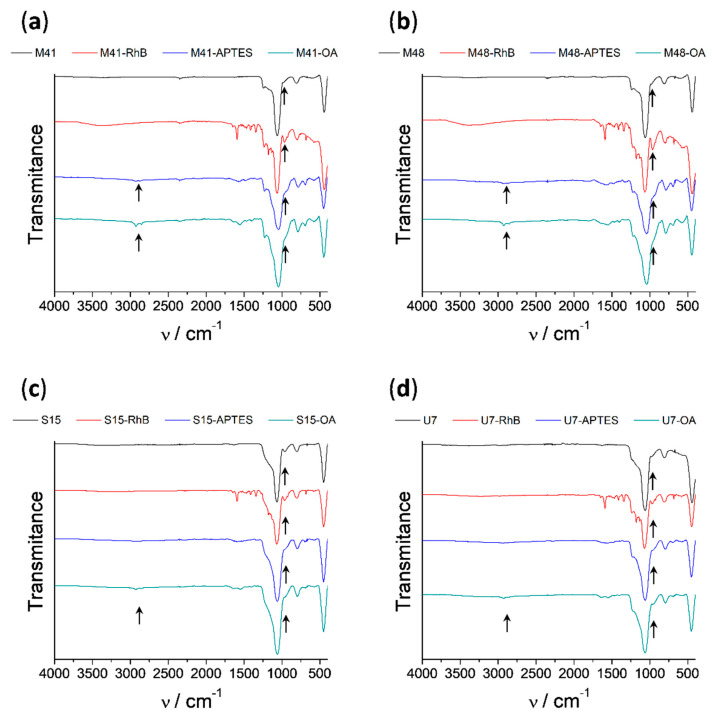
Infrared spectra of the consecutive steps of synthesis of the different solids: (**a**) M41, (**b**) M48, (**c**) S15 and (**d**) U7.

Infrared spectroscopy (FTIR) was also employed to follow the loading and functionalization processes leading to solids MSP-OA (see Figure 4). In all the FTIR spectra, the dominant bands are those due to the silica matrix (1060, 800 and 450 cm^−1^) giving rise to very similar spectra between them. However, some changes in minor bands can be related with the presence of specific organic groups in solids functionalized with APTES or OA. Consequently, it can be observed the decrease of the band around 950 cm^−1^ assignable to the silanol groups of the inorganic surface when the APTES group is anchored. Furthermore, it is visible the appearance of two bands at approximately 2900 cm^−1^ and 2850 cm^−1^ assignable to C–H bending vibrations. It can also be observed that these two bands increase as the amount of functionalized organic groups increase.

The contents of OA and RhB in MSP-OA solids were determined using TGA. TGA indicated an organic matter content of 72.75, 72.28, 133.64 and 46.14 mg RhB g^−1^ SiO_2_ in M41-OA, M48-OA, S15-OA and U7-OA respectively. The content of OA in the loaded and functionalized solids was 164.70, 119.86, 85.55 and 122.0 mg OA g^−1^ SiO_2_ in M41-OA, M48-OA, S15-OA and U7-OA respectively. The proportion in mmol per gram of SiO_2_ of all the moieties is summarized in Table 2.

### 3.2. Cargo Controlled Release

**Figure 5:**
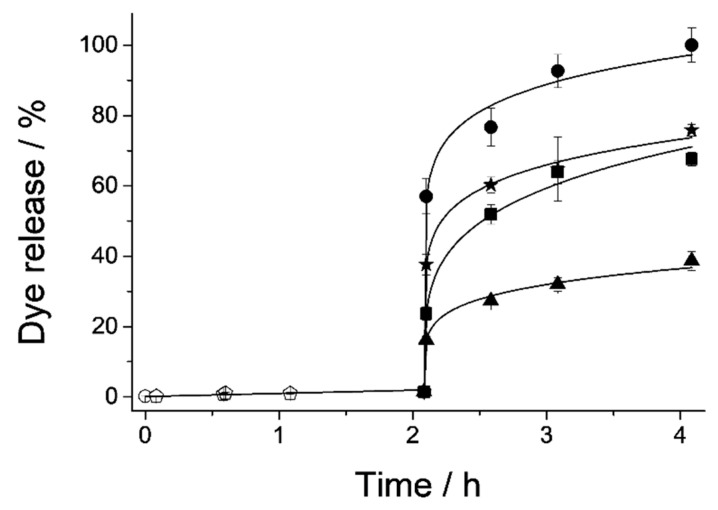
In vitro digestion of all the loaded and functionalized solids; saliva (○) and gastric (⬠) fluids show zero release for all the solids, and cargo release is produced when bile salts are present (▲ M41-OA, ■ S15-OA, ★ M48-OA, ● U7-OA).

**Figure 6:**
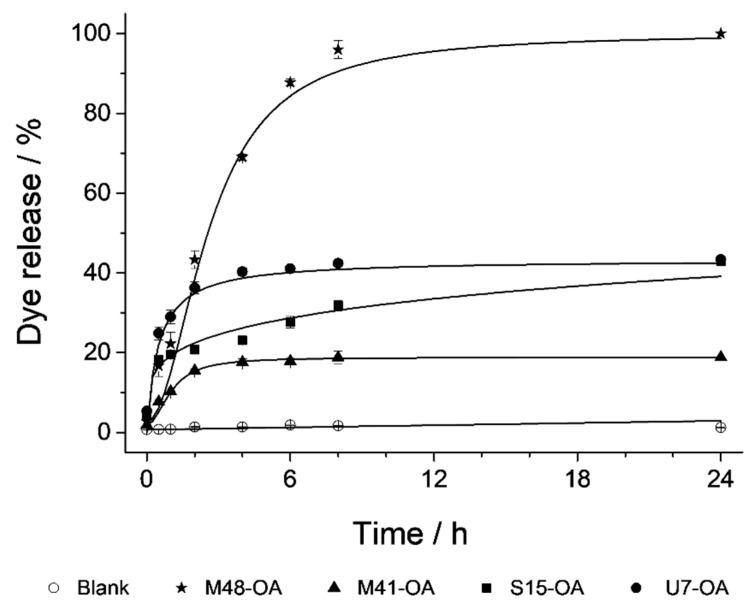
Release profile of RhB dye from all solids in a PBS suspension (○), showing zero release for all of them, and when bile extract is added (▲ M41-OA, ★ M48-OA, ■ S15-OA, ● U7-OA).

As stated above, the aim of this work was to test the ability of OA to maintain the cargo loaded into different MSP supports, and to ascertain the release profile of that cargo from the different supports under different conditions. In this section, several experiments were carried out with the objective of studying the bile-responsive controlled-release behavior of each capped material in detail. The performance of the microdevices along the GIT was evaluated in an in vitro digestion, where a batch assay was carried out simulating the digestive steps and conditions of the mouth, the stomach and the duodenum (Figure 5). In this experiment, the particles are subjected to changes in pH, salinity and biomolecules (including enzymes) as well as residence times in the different steps of the GIT. Maximum quantity of released cargo from each loaded and functionalized MSP-OA solid (μg RhB mg^−1^ MSP-OA) at each digestion step (saliva, gastric and duodenal fluids) is detailed in Table 3. From this experiment, we can conclude that cargo released in the first digestion steps (saliva and gastric fluid) is negligible compared with the signal appreciated in the first section of the small intestine, where the presence of the bile salts inhibits the hydrophobic interaction in the outer layer of the solid and provokes an important cargo release. Apart from the different behavior of each device, it can be derived that all of them can produce the release of the cargo in the first section of the small intestine where the bile salts appear (Figure 5). In order to remove the influence of the complex matrix conditions in the cargo release profiles, and also to verify the action of bile salts as triggering stimulus, a simplified release assay was carried out. Thus, delivery studies of all the loaded and functionalized solids (M41-OA, M48-OA, S15-OA and U7-OA) were performed in PBS medium and in the presence of bile salts, resulting in the cargo delivery, presenting each material a different release profile (Figure 6). Comparing both experiments, the release profile for each functionalized material along the two first hours of the in vitro digestion’s intestinal stage is similar to the one obtained during the delivery performed exclusively with bile extract and, hence, these data can be used as representative of the cargo release process for each material. Since the aim of this work was to maximize cargo delivery into the duodenum as the action place, the cargo release assay was restricted to the first two hours. However, since the action of bile salts is not exclusively reduced to the duodenum, but is prolonged to farthest sections of the small intestine (jejunum and/or ileum), the maximum amounts of released cargo from each MSP-OA system under the presence of bile extract after a 24 h action have also been included in Table 3. These data add extra information to the synthesized systems’ performance, indicating that a different behavior is observed for longer release times, which may be useful for other applications not contemplated in this work (such as (bio)molecules release in more distant absorption places of the small intestine or even in the large intestine).

### 3.3. Cargo Release Kinetics

In addition to achieving a notable cargo release triggered by the surfactant action of bile salts, it is also an important objective of this work to evaluate the ability of the studied systems to control the payload release, and distinguish whether it is bulk or sustained over time. For this purpose, the release kinetics data of the first 60% of the payload (data in Figure 6) were adjusted to four different mathematical models: zero order, first order, Higuchi and Korsmeyer–Peppas (K–P). All mathematical equations (Equations (1) – (4)) are detailed in Section 2.6 in Materials and Methods. In each of these models, certain premises are assumed, and different parameters are taken into account to describe the release process. The zero order model describes immediate and constant time release processes, usually of very soluble drugs in aqueous media. The first order model considers the change in concentration of the payload over time, associated with a decrease in the concentration of the drug in the initial support. Higuchi’s model, developed in the 1960s, marked a revolution in the modeling and understanding of drug release, since the matrix from which cargo is released was considered for the first time. This model is based on the premise of a uniform insoluble matrix with a cargo concentration much higher than the one released. Additionally, in this model, it is contemplated that the external medium penetrates to dissolve the loaded drug, which is unidirectional and constantly released. Finally, the semi-empirical model of K–P or “power law” consists of a modification of the Higuchi model with the aim of making it more general. It is applied for drug release from matrices in which several phenomena occur simultaneously, not only diffusion. Two parameters are obtained from the mathematical adjustment of the data to this equation: *K*, which is the constant related to the cargo’s diffusion velocity, and *n*, which is related to the type of diffusion and can be *n* = 0.45 in case of Fickian diffusion from cylindrical shaped pores, or 0.45 < *n* ≤ 0.89 if the diffusion is non-Fickian [51].

As it can be seen in Table 4, data fit correctly to more than one equation. However, the model that best explains all the data as a whole is K–P, since it assesses in its formula different types of diffusion from the matrix.

From the mathematical adjustment to this model the parameters *K, n* and *b* can be obtained (see Table 4). The values of these parameters allow us to better understand the process by which the cargo is released in the studied systems. If we compare *n*, which is forced by definition to be equal or bigger than 0.45 [53], it can be seen that from M41-OA, S15-OA and U7-OA the cargo is released by Fickian diffusion, as defined by the model for matrices with cylindrical structure. However, M48-OA releases the cargo with non-Fickian diffusion since the porous structure is not cylindrical but three-dimensional. On the other hand, the *y*-interception values correspond to the parameter *b*, which represents a phenomenon called "burst release" by which the load is immediately released in the medium with the triggered stimulus. Burst release is an unfavorable phenomenon in the processes of sustained release, but it is favorable in the present work since the pursued objective is the immediate payload release when the specific stimulus (bile salts) appears.

There are some articles that attempt to explain the mechanism of burst release [54], relating it to the structure of the guest molecules, their interaction with the host material, and especially with the structural characteristics of the support. The results obtained in this research are in agreement with data in the literature [33], where it was concluded that among different studied supports, the higher burst release was reached by the materials with textural-intraparticle pores in addition to the “surfactant-templated” mesopores, followed by the materials with larger mesopores, and finally those with smaller pore size.

The results of the kinetic models are related with those of the N_2_ adsorption-desorption isotherms (see Figure 3, bottom, and Table 1). It can be observed that the highest burst release is reached by the U7-OA system, which has completely filled mesopores and partially filled textural pores. Then, the next highest *b* value is achieved by the S15-OA system, since among the supports that only have “surfactant-templated” mesopores, S15 is the one with the largest pore size. Finally, although the solid M41 has a pore size slightly larger than M48, the complex internal structure of this last solid might be the cause of the difference between M41-OA and M48-OA burst release. Consequently, all the parameters were obtained, and their explanations lead to the conclusion that U7-OA is the system with the biggest payload release in the first hours of stimulus and with highest velocity. This explanation is also applicable to the obtained *K*-values. The cargo diffuses with greater velocity from the systems with textural-intraparticle pores (U7), then systems with large pores (S15), followed by systems with smaller pores (M41) and finally from porous matrices with complex structures (M48) (see Table 4).

### 3.4. In Vitro Biocompatibility Test: Interaction with Cells of the GIT

**Figure 7:**
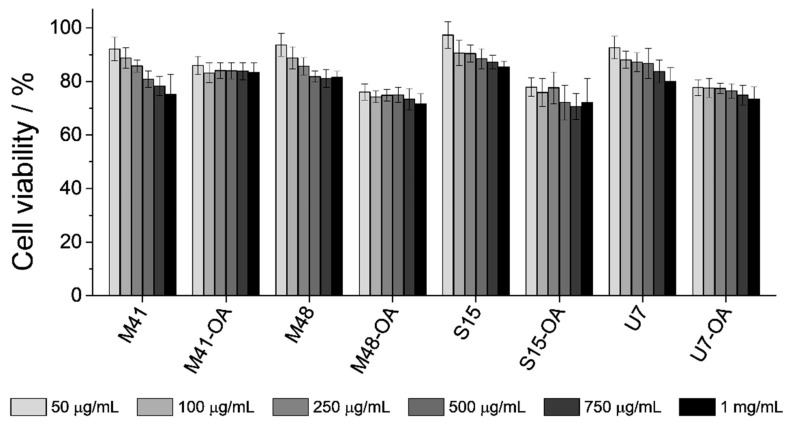
MTT cell viability assay. Caco-2 cells were treated with the bare MSPs and the final solids (MSPs-OA) at concentrations of 50, 100, 250, 500, 750 and 1000 μg/mL (increasing from light grey to black) for 24 h. After incubation, cell viability was quantified using MTT reagent and solubilization of formazan crystals into DMSO.

In addition to the cargo release experiments from the four different supports, it was also an objective of this study to assess the biocompatibility of the developed microsystems. Therefore, studies with all the bare MSPs and final loaded-and-functionalized solids (MSP-OA) were performed employing Caco-2 human colon adenocarcinoma cells in order to exclude any toxic effect of microparticles. Other studies in the literature reported low toxicity levels for similar mesoporous materials at low particle concentrations [55], reason why cells in this work where exposed to higher—even critical—particle concentrations. Thus, cells were treated with the corresponding system for 24 h at final concentrations of 50, 100, 250, 500, 750 and 1000 μg/mL. After this time, a cell viability assay using MTT was performed, which is based in the absorbance measurement of formazan produced by oxidoreductase enzymes of viable cells [56]. The formazan crystals generated are insoluble in the growing medium, so DMSO solvent must be added after medium-removal in order to solubilize the generated compound. The absorbance of the resulting purple solution was measured at λ_exc_ 550 nm and compared with the one achieved by control cells. The obtained results are depicted in Figure 7.

As it can be seen, MTT cell viability assay indicated that even at concentrations as high as 1 mg/mL the level of cell viability is quite high (approximately 85–75%). Although the functionalized particles are slightly more toxic than the starting MSPs, this difference is very low. Furthermore, unlike the bare starting materials, it can be seen that cell viability did not decrease by increasing particle concentration, even at values as high as 1 mg/mL. This fact might be related to the differences on particle stability in the culture medium. It has been reported in the literature that cell viability decreased when cell cultures were exposed to increasing particle concentrations, as these particles precipitate on the cell monolayer. However, stable suspensions with increasing concentration of the same particles maintained constant cell viability [57]. After this, it could be assumed that the functionalization of the MSPs with OA produces more stable suspensions and that, then, the increase of the particle concentration might not affect the cell-viability. In conclusion, the assay suggested that the developed systems loaded with RhB and functionalized with OA were well-tolerated by the cells.

### 3.5. In Vivo Pharmacokinetic Studies

The difficulty of many physiological factors (such as exact pH and composition of GIT fluids, enzymes, gastric emptying, GIT motility, etc.) to be incorporated into the in vitro models requires the use of in vivo models to evaluate the final performance of a dispositive when these factors are crucial. Therefore, to prove the specific opening of the gated material in the SI and quantify cargo release in plasma, in vivo pharmacokinetic studies were carried out. U7-OA solid was the chosen to be tested because, although its biocompatibility and ability to protect the cargo are the same as all the other solids, its payload release and velocity in the first section of the small intestine in simulated conditions (vide ante) are the highest ones. These reasons make of it an ideal system for immediate payload release in the duodenum after protecting the cargo from the adverse stomach conditions.

**Figure 8:**
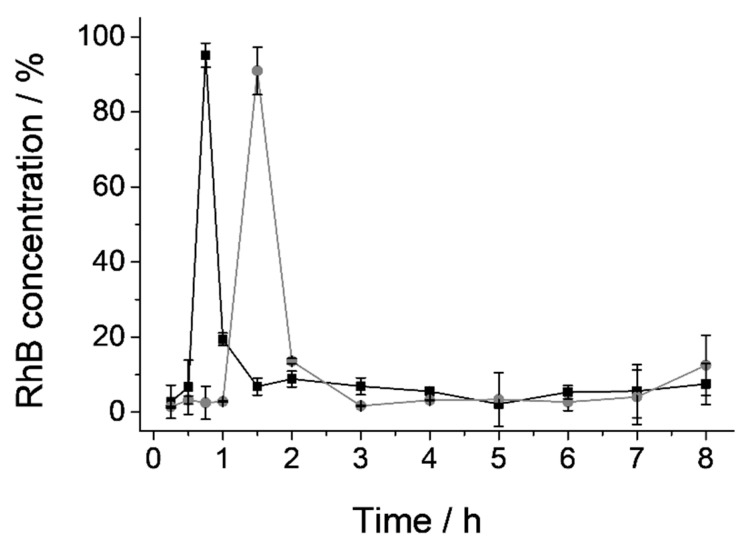
RhB concentration (%) in plasma for subjects of group 1 (RhB solution, black ■) and group 2 (U7-OA suspension, gray ●).

To perform the pharmacokinetic studies, male Wistar rats were divided into two groups (see Section 2.10 in Materials and methods). The subjects in group 1 were treated with 2 mL of RhB solution (0.84 mg/mL), and rats from group 2 were treated with 2 mL of a suspension of U7-OA microparticles (containing 150 mg of solid, which release 1.67 mg of RhB). RhB concentration in plasma as a function of time for both groups of rats is shown in Figure 8. As it could be seen, subjects from group 1 (that received the RhB solution) showed maximum concentration of the molecule (C_max_) at 45′ after administration (black line); however, rats in group 2 presented maximum RhB levels at 1.5 h (gray line). Due to the high permeability of RhB through the intestinal membrane, the delay of the maximum concentration of this molecule in plasma can be related to the retard of its delivery in the duodenum. Thus, in group 1, the RhB absorption is almost immediate after gastric emptying, while RhB encapsulated into the U7-OA microdevice will only be adsorbed after the bile salts have produced the opening of the microdevice and the dye release. Then, we can assume that the RhB is effectively encapsulated and protected during the initial steps of the GIT (i.e., the mouth and the stomach). This strategy can be useful for drug administration, in order to avoid stomach damage or degradation of the compound.

## 4. Conclusions

A set of different MSPs functionalized with oleic acid (MSP-OA) have been developed. The prepared materials are able to confine and protect their entrapped cargo in an aqueous environment and release it effectively only when bile salts are present. The release profiles of the different supports have been studied, emphasizing the role of the inorganic scaffold in active delivery regulated by oleic acid acting as a molecular gate. Thereby, the different behavior of the MSP-OA during the cargo delivery has been modeled using four different mathematical models: zero order, first order, Higuchi and Korsmeyer–Peppas (K–P). Among all, the K–P model gives the best adjustment for all solids and allows to distinguish between Fickian diffusion in solids M41-OA, S15-OA and U7-OA and the non-Fickian diffusion in M48-OA material. In order to test these MSP-OA materials as cargo release devices, in vitro cell viability experiments with Caco-2 adenocarcinoma cells were performed, showing that MSP-OA materials are non-toxic even at high particle concentrations. Based on the solids’ release kinetics, the U7-OA material was selected to essay duodenal bulk delivery in an in vivo model of Wistar rat. The results show a delay in the RhB absorption when using the U7-OA microdevice instead of the direct dosage of this model molecule. This delay indicates that the arrival of the RhB to the duodenum is retarded when using the U7-OA and corroborates the operation of the oleic acid functionalized MSPs. Payload discharge after gastric emptying achieved with this system stablishes an example of the possibility of using MSPs as protection and direct release agents into the duodenum. This fact makes of MSPs a good alternative to the existing administration methods employed until now.

## Figures and Tables

**Table 1:** BET specific surface values, pore volumes and pore sizes calculated from the N_2_ adsorption–desorption isotherms for selected materials.

	S_BET_ (m^2^ g^−1^)	Pore Volume (cm^3^ g^−1^)	Pore Size ^c^ (nm)
M41	1074.0	1.0	3.0
M41-OA	2.4	0.0	-
M48	1355.4	1.0	2.3
M48-OA	0.1	0.0	-
S15	670.7	0.8	5.4
S15-OA	31.3	0.0	-
U7	922.1	0.7 ^a^ (0.8) ^b^	2.6 ^a^ (32) ^b^
U7-OA	96.4	0.07 ^a^ (0.4) ^b^	- ^a^ (34) ^b^

^a^ Pore volumes and pore sizes corresponding to “surfactant-templated” mesopores. ^b^ Pore volumes and pore sizes corresponding to textural intraparticle porosity. ^c^ Pore size was estimated using the BJH model applied on the adsorption branch of the isotherm.

**Table 2:** Content (α) in mmol g^−1^ SiO_2_ of RhB, APTES and OA in the loaded and functionalized solids (MSP-OA).

Solid	α_RhB_	α_APTES_	α_OA_
M41-OA	0.15	6.30	0.59
M48-OA	0.15	6.17	0.43
S15-OA	0.28	3.57	0.30
U7-OA	0.10	3.59	0.44

**Table 3:** Maximum quantity of released cargo from each MSP-OA solid (μg RhB mg^−1^ MSP-OA) in each digestion fluid (saliva, gastric and duodenal) compared to the maximum quantity reached in the cargo release assay using bile extract exclusively for 24 h.

Digestive fluid	M41-OA	M48-OA	S15-OA	U7-OA
Saliva	0.01 ± 3·10^−4^	0.02 ± 9·10^−4^	0.01 ± 7·10^−4^	0.01 ± 4·10^−4^
Gastric	0.10 ± 0.01	0.16 ± 0.01	0.16 ± 0.02	0.07 ± 0.02
Duodenal	3.35 ± 0.23	6.58 ± 0.15	5.86 ± 0.16	8.68 ± 0.39
Bile extract 24 h	4.84 ± 0.24	25.71 ± 0.38	11.02 ± 0.33	11.15 ± 0.20

**Table 4:** Parameters and coefficients of determination (r^2^) obtained by adjusting the data to each model Equations (1)–(4).

System	Zero Order Equation (1)		First Order Equation (2)			Higuchi Equation (3)		Korsmeyer–Peppas Equation (4)			
	*k*_0_ (min)	r^2^	*k*_1_ (min)	*C*_0_	r^2^	*K_H_* (min^−½^)	r^2^	*K* (min^−n^)	*n*	*b*	r^2^
M48-OA	0.3	0.98	0.0	7.4	0.7	4.01	0.97	0.99	0.77	1.76	0.995
M41-OA	0.1	0.8	0.0	4.3	0.6	1.25	0.97	1.52	0.45	0.71	0.97
S15-OA	0.1	0.6	0.0	9.2	0.5	1.8	0.8	1.71	0.45	5.50	0.87
U7-OA	0.2	0.7	0.0	27.9	0.5	3.08	0.90	3.21	0.45	6.36	0.92

## References

[1] Pérez-Esteve É., Ruiz-Rico M., De La Torre C., Llorca E., Sancenón F., Marcos M.D., Amorós P., Guillem C., Martínez-Máñez R., Barat J.M. (2016). Stability of different mesoporous silica particles during an in vitro digestion. Microporous Mesoporous Mater..

[2] Di Pasqua A.J., Sharma K.K., Shi Y.L., Toms B.B., Ouellette W., Dabrowiak J.C., Asefa T. (2008). Cytotoxicity of mesoporous silica nanomaterials. J. Inorg. Biochem..

[3] Izquierdo-Barba I., Colilla M., Manzano M., Vallet-Regí M. (2010). In vitro stability of SBA-15 under physiological conditions. Microporous Mesoporous Mater..

[4] Arruebo M. (2012). Drug delivery from structured porous inorganic materials. Wiley Interdiscip. Rev. Nanomed. Nanobiotechnol..

[5] Vallet-Regí M., Balas F., Arcos D. (2007). Mesoporous materials for drug delivery. Angew. Chem. Int. Ed..

[6] Descalzo A.B., Martínez-Máñez R., Sancenón F., Hoffmann K., Rurack K. (2006). The supramolecular chemistry of organic-inorganic hybrid materials. Angew. Chem. Int. Ed..

[7] Shang L., Bian T., Zhang B., Zhang D., Wu L.Z., Tung C.H., Yin Y., Zhang T. (2014). Graphene-supported ultrafine metal nanoparticles encapsulated by mesoporous silica: Robust catalysts for oxidation and reduction reactions. Angew. Chem. Int. Ed..

[8] Taguchi A., Schüth F. (2005). Ordered Mesoporous Materials in Catalysis. Microporous Mesoporous Mater..

[9] Angelos S., Liong M., Choi E., Zink J.I. (2008). Mesoporous silicate materials as substrates for molecular machines and drug delivery. Chem. Eng. J..

[10] García-Fernández A., Aznar E., Martínez-Máñez R., Sancenón F. (2020). New advances in in vivo applications of gated mesoporous silica as drug delivery nanocarriers. Small.

[11] Barbé C., Bartlett J., Kong L., Finnie K., Lin H.Q., Larkin M., Calleja S., Bush A., Calleja G. (2004). Silica particles: a novel drug‐delivery system. Adv. Mater..

[12] Llopis-Lorente A., Lozano-Torres B., Bernardos A., Martínez-Máñez R., Sancenón F. (2017). Mesoporous silica materials for controlled delivery based on enzymes. J. Mater. Chem. B.

[13] Mas N., Arcos D., Polo L., Aznar E., Sánchez-Salcedo S., Sancenón F., García A., Marcos M.D., Baeza A., Vallet-Regí M. (2014). Towards the development of smart 3D “gated scaffolds” for on-Command delivery. Small.

[14] Santos-Figueroa L.E., Giménez C., Agostini A., Aznar E., Marcos M.D., Sancenón F., Martínez-Máñez R., Amorós P. (2013). Selective and sensitive chromofluorogenic detection of the sulfite anion in water using hydrophobic hybrid organic-Inorganic silica nanoparticles. Angew. Chem. Int. Ed..

[15] Poyatos-Racionero E., Ros-Lis J.V., Vivancos J.-L., Martínez-Máñez R. (2018). Recent advances on intelligent packaging as tools to reduce food waste. J. Clean. Prod..

[16] Coll C., Casasús R., Aznar E., Marcos M.D., Martínez-Máñez R., Sancenón F., Soto J., Amorós P. (2007). Nanoscopic hybrid systems with a polarity-controlled gate-Like scaffolding for the colorimetric signalling of long-chain carboxylates. Chem. Commun..

[17] Pérez-Esteve E., Bernardos A., Martínez-Máñez R., Barat J.M. (2013). Nanotechnology in the development of novel functional foods or their package. an overview based in patent analysis. Recent Pat. Food Nutr. Agric..

[18] Lamprecht A., Schäfer U., Lehr C.-M.M. (2001). Size-Dependent bioadhesion of micro- and nanoparticulate carriers to the inflamed colonic mucosa. Pharm. Res..

[19] De Luis B., Llopis-Lorente A., Rincón P., Gadea J., Sancenón F., Aznar E., Villalonga R., Murguía J.R., Martínez-Máñez R. (2009). An interactive model of communication between abiotic nanodevices and microorganisms. Angew. Chemie-Int. Ed..

[20] Bernardos A., Marina T., Žáček P., Pérez-Esteve É., Martínez-Mañez R., Lhotka M., Kouřimská L., Pulkrábek J., Klouček P. (2015). Antifungal effect of essential oil components against Aspergillus niger when loaded into silica mesoporous supports. J. Sci. Food Agric..

[21] Barat J., Pérez-Esteve É., Bernardos A., Martínez-Mañez R. (2011). Nutritional effects of folic acid controlled release from mesoporous materials. Procedia Food Sci..

[22] Ruiz-Rico M., Daubenschüz H., Pérez-Esteve É., Marcos M.D., Amorós P., Martínez-Máñez R., Barat J.M. (2016). Protective effect of mesoporous silica particles on encapsulated folates. Eur. J. Pharm. Biopharm..

[23] Bernardos A., Piacenza E., Sancenón F., Hamidi M., Maleki A., Turner R.J., Martínez-Máñez R. (2019). Mesoporous Silica-Based Materials with Bactericidal Properties. Small.

[24] Awaad A., Nakamura M., Ishimura K. (2012). Imaging of size-dependent uptake and identification of novel pathways in mouse Peyer’s patches using fluorescent organosilica particles. Nanomed. Nanotechnol. Biol. Med..

[25] Hussain N., Jaitley V., Florence A.T. (2001). Recent advances in the understanding of uptake of microparticulates across the gastrointestinal lymphatics. Adv. Drug Deliv. Rev..

[26] Fu C., Liu T., Li L., Liu H., Chen D., Tang F. (2013). The absorption, distribution, excretion and toxicity of mesoporous silica nanoparticles in mice following different exposure routes. Biomaterials.

[27] Alothman Z.A. (2012). A review: Fundamental aspects of silicate mesoporous materials. Materials (Basel).

[28] Asefa T., Tao Z. (2012). Mesoporous silica and organosilica materials-Review of their synthesis and organic functionalization. Can. J. Chem..

[29] Li Y., Li N., Pan W., Yu Z., Yang L., Tang B. (2017). Hollow mesoporous silica nanoparticles with tunable structures for controlled drug delivery. ACS Appl. Mater. Interfaces.

[30] Legnoverde M.S., Basaldella E.I. (2016). Influence of particle size on the adsorption and release of cephalexin encapsulated in mesoporous silica SBA-15. Mater. Lett..

[31] Popova M., Szegedi A., Mavrodinova V., Novak Tušar N., Mihály J., Klébert S., Benbassat N., Yoncheva K. (2014). Preparation of resveratrol-loaded nanoporous silica materials with different structures. J. Solid State Chem..

[32] Martín A., Morales V., Ortiz-Bustos J., Pérez-Garnes M., Bautista L.F., García-Muñoz R.A., Sanz R. (2018). Modelling the adsorption and controlled release of drugs from the pure and amino surface-functionalized mesoporous silica hosts. Microporous Mesoporous Mater..

[33] Pérez-Esteve É., Ruiz-Rico M., De La Torre C., Villaescusa L.A., Sancenón F., Marcos M.D., Amorós P., Martínez-Máñez R., Barat J.M. (2016). Encapsulation of folic acid in different silica porous supports: A comparative study. Food Chem..

[34] Silveira G.Q., Da Silva R.S., Franco L.P., Vargas M.D., Ronconi C.M. (2015). Redox-responsive nanoreservoirs: The effect of different types of mesoporous silica on the controlled release of doxorubicin in solution and in vitro. Microporous Mesoporous Mater..

[35] Wang S. (2009). Ordered mesoporous materials for drug delivery. Microporous Mesoporous Mater..

[36] Donner T., Sarkar S. (2000). Insulin–Pharmacology, Therapeutic Regimens, and Principles of Intensive Insulin Therapy.

[37] Lu P.J., Hsu P.I., Chen C.H., Hsiao M., Chang W.C., Tseng H.H., Lin K.H., Chuah S.K., Chen H.C. (2010). Gastric juice acidity in upper gastrointestinal diseases. World J. Gastroenterol..

[38] Ulker I., Yildiran H. (2019). The effects of bariatric surgery on gut microbiota in patients with obesity: A review of the literature. Biosci. Microbiota Food Heal..

[39] Sardo P., Walker J.H. (2008). Bariatric surgery: Impact on medication management. Hosp. Pharm..

[40] De Geraldo M.S.P., Fonseca F.L.A., de Veiga Gouveia M.R.F., Feder D. (2014). The use of drugs in patients who have undergone bariatric surgery. Int. J. Gen. Med..

[41] Kurlan R., Nutt J.G., Woodward W.R., Rothfield K., Lichter D., Miller C., Carter J.H., Shoulson I. (1988). Duodenal and gastric delivery of levodopa in parkinsonism. Ann. Neurol..

[42] Lundqvist C. (2007). Continuous levodopa for advanced Parkinson’s disease. Neuropsychiatr. Dis. Treat..

[43] Bredberg E., Nilsson D., Johansson K., Aquilonius S.M., Johnels B., Nyström C., Paalzow L. (1993). Intraduodenal infusion of a water-Based levodopa dispersion for optimisation of the therapeutic effect in severe Parkinson’s disease. Eur. J. Clin. Pharmacol..

[44] Antonini A., Odin P. (2009). Pros and cons of apomorphine and l-dopa continuous infusion in advanced Parkinson’s disease. Park. Relat. Disord..

[45] Santos-García D., de Deus T., López-Pazos E., Macías-Arribi M., Llaneza-González M.A., de la Fuente-Fernández R., Echarri-Piudo A., Carpintero P. (2014). Management of complications related to intraduodenal infusion of levodopa/carbidopa in patients with Parkinson’s disease. Rev. Neurol..

[46] Poyatos-Racionero E., Pérez-Esteve É., Dolores Marcos M., Barat J.M., Martínez-Máñez R., Aznar E., Bernardos A. (2019). New Oleic Acid-Capped Mesoporous Silica Particles as Surfactant-Responsive Delivery Systems. ChemistryOpen.

[47] Cabrera S., El Haskouri J., Guillem C., Latorre J., Beltrán-Porter A., Beltrán-Porter D., Marcos M.D., Amorós P. (2000). Generalised syntheses of ordered mesoporous oxides: The atrane route. Solid State Sci..

[48] Meléndez-Ortiz H.I., Perera-Mercado Y.A., García-Cerda L.A., Mercado-Silva J.A., Castruita G. (2014). Influence of the reaction conditions on the thermal stability of mesoporous MCM-48 silica obtained at room temperature. Ceram. Int..

[49] Zhao D., Feng J., Huo Q., Melosh N., Fredrickson G.H., Chmelka B.F., Stucky G.D. (1998). Triblock Copolymer Syntheses of Mesoporous Silica with Periodic 50 to 300 Angstrom Pores. Science.

[50] El Haskouri J., de Zárate D.O., Guillem C., Latorre J., Caldés M., Beltrán A., Beltrán D., Descalzo A.B., Rodríguez-López G., Martínez-Máñez R. (2002). Silica-Based powders and monoliths with bimodal pore systems. Chem. Commun..

[51] Bruschi M.L. (2015). Mathematical models of drug release. Strategies to Modify the Drug Release from Pharmaceutical Systems.

[52] Versantvoort C.H.M., Oomen A.G., Van De Kamp E., Rompelberg C.J.M., Sips A.J.A.M. (2005). Applicability of an in vitro digestion model in assessing the bioaccessibility of mycotoxins from food. Food Chem. Toxicol..

[53] Ritger P.L., Peppas N.A. (1987). A simple equation for description of solute release I. Fickian and non-fickian release from non-swellable devices in the form of slabs, spheres, cylinders or discs. J. Control. Release.

[54] Huang X., Brazel C.S. (2001). On the importance and mechanisms of burst release in matrix-Controlled drug delivery systems. J. Control. Release.

[55] Choi S.J., Kim Y.R. (2013). Bioinspired layered nanoclays for nutraceutical delivery system. ACS Symp. Ser..

[56] Sgouras D., Duncan R. (1990). Methods for the evaluation of biocompatibility of soluble synthetic polymers which have potential for biomedical use: 1-Use of the tetrazolium-based colorimetric assay (MTT) as a preliminary screen for evaluation of in vitro cytotoxicity. J. Mater. Sci. Mater. Med..

[57] Yazdimamaghani M., Barber Z.B., Hadipour Moghaddam S.P., Ghandehari H. (2018). Influence of Silica Nanoparticle Density and Flow Conditions on Sedimentation, Cell Uptake, and Cytotoxicity. Mol. Pharm..

